# Supplementary material for: Measuring data rot: An analysis of the continued availability of shared data from a Single University
Source: PLoS One. 2024 Jun 5;19(6):e0304781. doi: 10.1371/journal.pone.0304781 (PMC11152257; doi:10.1371/journal.pone.0304781)
Supplement: Attachment — Submitted filename: DataRot_ResponseToReviewers.docx [file pone.0304781.s001.docx]

To the reviewers,

Thank you so much for the informative feedback on the submitted version of my manuscript. I have attempted to address all of your comments and have listed the updates here. Due to the significant number of changes, they are roughly listed according to the article section in which they appear for ease of tracking:

Introduction

- I separated the introduction and the literature review.
- I added definitions of “supplemental data link” and “supplementary information”. I have tried to consistently use these two terms throughout the manuscript.

Literature review

- I added references to work done by Herbert Van de Sompel and Martin Klein on reference rot and content drift, alongside a recently published article about the preservation of published articles by Martin Eve.
- I added reference to Pepe, et al. article about availability of data from astronomy articles. I also cited data from this article in the discussion for comparison to findings in this analysis.
- I clarified which Borgman and Tenopir citations support the claim for making researchers more likely to share data.

Methodology

- I added information on the process by which we search for Caltech-authored articles to add to the institutional repository.
- I added information as to why we stopped curation supplemental data links in 2022 (to prepare for a repository upgrade in 2023).
- I added information on the disciplinary breakdown of Caltech-authored articles, pulling data from Web of Science.
- I sampled article metadata from the institutional repository and compared this information in the published articles to gather an estimate of the accuracy and completeness of the collection of supplemental data links used in this analysis. Sampling information was reported in the results section. Findings impacted my error calculations and the discussions about supplementary information and PDF links throughout the article.
- I added citations to the R packages as references. I removed the mention of the R package “utils” as I had removed use of that from the final code.

Results

- I added a note about the one article in the dataset that contains 28 links to supplemental data (most of the links go to individual structure accessions in Protein Data Bank).
- I changed the x-axes of both figures to show the age of the article rather than the year published, so that newer articles are now on the left and older articles are on the right.
- I flipped the y-axis on figure 2 by plotting the availability of data rather than the unavailability for consistency with corresponding studies.
- I updated the fit model for figure 2 to be a Poisson regression, as was done in a corresponding study by Vines, et al. This fit includes a confidence interval and p-value.
- I redid all of the error calculations, fit modelling, and figures in R so that everything is reproducible and transparent.

Discussion

- I updated the discussion about limitations of the dataset to better reflect the results of sampling the quality of the metadata in the repository.
- I added a reference to work by Van de Sompel, et al. showing that researchers use URLs instead of DOIs when citing scholarly articles.
- I added a recommendation that journals should shift away from supplementary information in PDFs in favor of having researcher put data in to a data repository.
- I did not do an analysis of the disciplines of data in the institutional data repository, CaltechDATA, to see where it might be filling gaps in the landscape of disciplinary repositories. This is beyond the scope of this article.

Data availability

- I updated the links to the shared data and code as the repository generates new DOIs for each version of the content.
