# Supplementary material for: Measuring data rot: An analysis of the continued availability of shared data from a Single University
Source: PLoS One. 2024 Jun 5;19(6):e0304781. doi: 10.1371/journal.pone.0304781 (PMC11152257; doi:10.1371/journal.pone.0304781)
Supplement: Attachment — Submitted filename: DataRot_ResponseToReviewers.docx [file pone.0304781.s002.docx]

To the reviewers,

Thank you so much for the informative feedback on the submitted version of my manuscript. I have addressed all of the comments and have listed the updates here. The changes are as follows, with the request and how I modified the manuscript in the subsequent bullet point:

Request: P.3, Line 43: text should be “It can BE difficult…”

- I updated the text as requested.

Request: P.3, Line 50: the text in parantheses should be a new sentence

- I made the parenthetical into a new sentence.

Request: P.11, Line 215: same problem as above

- I updated the parenthetical to be part of the same sentence following a semicolon.

Request: P.12, Line 222: a reference is needed for this claim: “more publishers requesting that authors use data availability statements in articles instead of noting shared data as a citation or footnote.” Can the authors cite a study that shows that publishers request the use of data availability statements more often than requesting data citations?

- I removed the following portion of the text – “instead of noting shared data as a citation or footnote” – and added citations to show the increase in published data availability statements. This update better reflects the point I’m making about increased data availability statements (as opposed to the explicit method of how data is shared in articles).

Request: P. 13, Line 242: Should it be clarified that the data curation error rate is calculated only based on the sample?

- I edited the sentence to reflect the calculated error rate is based on sampling.
